# Effects of Huangqi Gancao Decoction on intestinal immunity and microbiota in immunocompromised mice models

**DOI:** 10.3389/fphar.2024.1390170

**Published:** 2024-05-01

**Authors:** Hai Zhou, Jianpeng Yan, Ke Zhou, Peng Ji, Yanming Wei, Yongli Hua

**Affiliations:** ^1^ Tranditional Chinese Veterinary Medicine Laboratory, College of Veterinary Medicine, Gansu Agricultural University, Lanzhou, Gansu, China; ^2^ Lanzhou Animal Disease Control Center, Lanzhou, Gansu, China

**Keywords:** Huangqi Gancao Decoction, immunosuppression, CD4^+^CD8^+^ double-positive T lymphocytes, intestinal intraepithelial lymphocytes, microbiota

## Abstract

**Background:**

The classical medicinal formula Huangqi Gancao Decoction (HQGCD), originating from the medical book" *Yi Lin Gai Cuo*". Up to now, the studies focusing on the immunoenhancement effects of HQGCD are few, and the actionpathway is not yet clear.

**Method:**

In this study, SPF male KM mice were utilized as a model for immunosuppression. Comprehensive observations were made regarding the general behavior and condition of the mice, in addition to monitoring fluctuations in body weight and food intake. The blood routine index was measured, and morphological changes in the ileum and colon tissues were examined. The level of secretory immunoglobulin A (sIgA), superoxide dismutase (SOD), and malondialdehyde (MDA) in ileum and colon tissues were quantified. Additionally, the bone marrow total DNA index was assessed. Flow cytometry analyzed the proportions of CD3⁺, CD4⁺, CD8⁺, and CD4^+^CD8^+^ double-positive (DP) T lymphocytes in small intestinal intraepithelial lymphocytes (IELs). Lastly, the composition and diversity of the cecal microbiota were evaluated using 16S rDNA sequencing technology.

**Results:**

After HQGCD intervention, there were no significant changes in the mice’s feed intake and body weight. However, the tissue structures of the ileum and colon showed recovery. In the blood routine index, there was an increase in the total white blood cell count, lymphocyte count, red blood cell count, hematocrit, and hemoglobin content. Additionally, the bone marrow total DNA index was elevated. Level of SOD and sIgA in ileum and colon tissues increased, while the level of MDA decreased. The proportions of CD3⁺ and CD4⁺ T lymphocytes within IELs increased, along with an increase in DP T lymphocytes in IELs (DP IELs), whereas the proportion of CD8⁺ T lymphocytes decreased. The cecal microbiota underwent changes, with an increase in the variety and number of beneficial microbiota.

**Conclusion:**

HQGCD could restore the intestinal immune function of immunocompromised mice, and had a certain positive effect on cecal microbiota.

## 1 Introduction

Immunosuppression is the reduction or impairment of the immune system’s function in humans or animals, leading to diminished resistance to pathogens and other harmful substances and an increased vulnerability to diseases (Tami et al., 1986). The intestinal immune system, through its complex and fine network structure, constitutes the body’s first line of defense against foreign pathogens. It can recognize and eliminate invading microorganisms, distinguish between beneficial and harmful bacteria, and regulate the body’s immune response to these microorganisms. When immunosuppression occurs in the body, the immune function of the intestinal is also affected.

HQGCD primary indications include the treatment of painful urination and urinary incontinence in the elderly, demonstrating significant efficacy irrespective of disease duration (Lan et al., 2024). HQGCD can treat prostatitis, genital warts, chronic hepatitis B, liver cirrhosis, alcoholic liver fibrosis and diabetes (Zou, 1990; Chen, 2005). Both Astragali Radix (*Astragalus membranaceus* (Fisch.) Bunge) and Glycyrrhizae Radix et Rhizoma (*Glycyrrhiza uralensis* Fisch.) have the effect of tonifying “middle-Jiao and Qi” in this decoction, the majority of studies on HQGCD have concentrated on its efficacy in treating urinary tract diseases and hepatopathy, with a notable scarcity of contemporary pharmacological research investigating its mechanisms of immune modulation.

Cyclophosphamide (Cy), a widely used broad-spectrum anti-tumor drug, exhibits potent cytotoxic effects, particularly inhibiting rapidly proliferating tissues like bone marrow and lymphoid tissue, thereby reducing the body’s immunity (Yu et al., 2018; Liu et al., 2019; Khan et al., 2022). Researchers frequently employ Cy to create immunocompromised models. Cy induces bone marrow suppression, impairing its hematopoietic function. Consequently, this reduces the number of white and red blood cells in the bloodstream, a condition referred to as “Blood Deficiency” in traditional Chinese medicine. The intestinal microbiota forms a complex microbial ecosystem, with its species composition and population maintained within a dynamic equilibrium, fostering mutually beneficial relationships with the host as an essential bodily component (Adak and Khan, 2019). These microbiotas interact closely with the host, for instance, in response to cold conditions, the intestinal microbiota in mice can enhance nutrient absorption by increasing the length of the intestinal villi and microvilli, thus meeting the increased energy demands (Chevalier et al., 2015). Conversely, non-specific gastrointestinal complications often arise during high-altitude travel, attributed to alterations in the intestinal microbiota driven by reduced atmospheric oxygen pressure (Adak et al., 2013). When intestinal immunity is compromised, the diversity of the intestinal microbiota undergoes changes, characterized by a decrease in the richness of beneficial microorganisms and an increase in the richness of pathogenic microorganisms (Ying et al., 2020).

In this study, mice were injected intraperitoneally with Cy to establish models of immunosuppression. They were then treated with HQGCD to explore its therapeutic effects and mechanisms on intestinal immunosuppression and its impact on the cecum microbiota. This research aims to provide a theoretical basis for the future development and application of HQGCD.

## 2 Materials and methods

### 2.1 Reagents and instruments

*A*.* membranaceus* and *G. uralensis* decoction pieces were purchased from the Yellow River medicinal materials market in Lanzhou, and identified by Prof. Yan-ming Wei from the department of Chinese veterinary medicine, college of veterinary medicine, Gansu Agricultural University. Cyclophosphamide (lot number: 22111425) was purchased from Jiangsu Hengrui Pharmaceutical Co., LTD; DTT (Cat. No.: 3483-12-3), EDTA (Cat. No.: 60-00-4), Percoll (Cat. No.: 65455-52-9) and BCA protein assay kit (Cat. No.: PC0020) were purchased from Beijing Solaibao Technology Co., Ltd; SIgA ELISA kit (Cat. No.: ml001917) was purchased from Shanghai Enzyme-linked Biotechnology Co., Ltd.; MDA Biochemistry Kit (Cat. No.: A003-1) and SOD Biochemistry Kit (Cat. No.: A001-3) were purchased from Nanjing Jiancheng Institute of Bioengineering; PE/Cyanine5 Anti-Mouse CD3 Antibody (Cat. No.: E-AB-F1013G) was purchased from Wuhan elabscience Biotechnology Co., Ltd.; CD4 Monoclonal Antibody (GK1.5), FITC (Cat. No.: 11-0041-81) and CD8a Monoclonal Antibody (53-6.7), PE (Cat. No.: 12-0081-82) were purchased from eBioscience, Methanol, acetonitrile (chromatography pure, Tianjin Damao Chemical Reagent Factory); phosphoric acid (Chromatography pure, Tianjin Kemio Chemical Reagent Co., LTD.); Calycosin (C_16_H_12_O_5_, MW = 284.26, CAS: 20575-57-9), Glycyrrhizic acid (C42H62O16, MW = 822.92, CAS: 1405-86-3), were purchased from Nanjing Yuanzhi Biotechnology Co., LTD, Inc, etc.

RE-6000 rotary evaporator (Shanghai Yarong Biochemical Instrument Factory); Vacuum freeze dryer (Shanghai Yuming Instrument Co., LTD.); TG16 desktop high-speed centrifuge (Shanghai Luxiangyi Centrifuge Instrument Co., LTD.); High-speed refrigerated centrifuge (HITACHI, Japan); Leica Paraffin rotary microtome (Leica Company, United States); High-throughput tissue grinding instrument SCIENTZ-48L (Ningbo Xinzhi Biological Co., LTD.); Agilent Technologies 1260 high performance liquid chromatography (Agilent, United States); Ultraviolet spectrophotometer (Shanghai Lang Gan Experimental Equipment Co., LTD.); SPectra Max Plus384 microplate reader (Meigu Molecular Instruments Co., LT.

### 2.2 The preparation of HQGCD

120 g *A. membranaceus* decoction pieces and 24 g *G. uralensis* decoction pieces were prepared, maintaining a 1:10 ratio with distilled water. The mixture was brought to a boil over high heat, and then simmered on low heat for 30 min. The residue was re-extracted with eight times the volume of distilled water using the same boiling and simmering process. The filtrates from both extractions were combined, concentrated, and freeze-dried to obtain the dry powder of HQGCD.

### 2.3 HPLC determination of the active metabolites in HQGCD

#### 2.3.1 Chromatographic conditions

The chromatographic conditions were as followes: Agilent Zorbax-SB C18 chromatographic column (250 mm × 4.6 mm, 5 μm), the mobile phase consisted of 0.1% phosphoric acid aqueous solution (A) - acetonitrile (C) with a gradient elution: 0–5 min, 30% C; 5–8 min, 35% C; 8–13 min, 40% C; 13 min, 45% C; flow rate: 0.8 mL/min; detection wavelength: 280 nm; injection volume: 20 μL; column temperature: 29°C.

#### 2.3.2 The preparation of HQGCD samples

100 mg of HQGCD freeze-dried powder was accurately weighed, and the methanol was added to a constant volume of 10 mL, sonicated for 10 min, and the supernatant was filtered with 0.22 μm microporous filter membrane to be tested.

#### 2.3.3 The preparation of standard solution

24 and 20 mg of standard samples of calycosin and glycyrrhizic acid were accurately weighed, and the methanol was added to a constant volume of 10 mL, sonicated for 2 min, and the supernatant was filtered with 0.22 μm microporous filter membrane to be tested.

#### 2.3.4 HPLC determination

According to the chromatographic conditions of “2.3.1”, the standard solution (20 μL) and HQGCD samples (20 μL) were respectively taken and determined by HPLC.

### 2.4 Animal experiments

40 SPF male KM mice, weighing 22 ± 2 g and aged 6 weeks, were obtained from the Laboratory Animal Center, Lanzhou Veterinary Research Institute, Chinese Academy of Sciences (Certificate No.: SCXX (Gan) 2023-0002). The mice were housed under controlled conditions with a standard diet and water, at temperatures ranging from 20°C to 25°C, humidity level between 30% and 39%, and a 12-h day-night cycle. These mice were randomly divided into five groups: Control group (CON), Model group (MOD), and three HQGCD dose groups (high dose: HQGCD-H, 0.84 g/kg; medium dose: HQGCD-M, 0.42 g/kg; low dose: HQGCD-L, 0.21 g/kg). To establish models of immunosuppression, MOD and HQGCD were administered intraperitoneal injections of Cy at 80 mg/kg on the first 3 days. In contrast, CON received an equivalent volume of normal saline. From the fourth to the tenth day, three dose groups were treated with their respective doses via gavage, while CON and MOD were given the same volume of normal saline once daily. 24 h after the final administration of HQGCD, samples, including blood, femurs, ileum, colon, and cecum contents, were collected from all mice for subsequent analysis.

### 2.5 General behavioral observations

During the modeling and administration of each group, their mental state and planta pedis color were observed daily.

### 2.6 Body weight and feed intake

During the experiment, each group’s body weight and feed intake were weighed and recorded daily.

### 2.7 Histological and morphological observation of ileum and colon sections

The harvested ileum and colon were fixed in formalin solution, embedded in paraffin, stained with HE, and then observed under a light microscope and imaged with Case Viewer software.

### 2.8 Blood routine index

Blood samples were collected into tubes containing EDTA as an anticoagulant. The total white blood cell count, lymphocyte count, total red blood cell count, hematocrit, and hemoglobin content were then measured using a hematology analyzer.

### 2.9 Bone marrow total DNA index

Femurs were extracted from the left legs of mice, with the femoral ends subsequently punctured to facilitate marrow extraction. The bone marrow was then eluted into centrifuge tubes using 0.005 mol/L CaCl_2_, followed by immediate cooling to maintain cellular integrity before centrifugation. After removal of the supernatant, the pellet was resuspended in 0.2 mol/L HClO_4_ for a series of steps, including suspension, precipitation, incubation in a water bath, cooling, and subsequent centrifugation. The supernatant’s optical density (OD) was measured at a wavelength of 268 nm using an ultraviolet spectrophotometer (Liao et al., 1998). This OD value was normalized to the mass of the corresponding left femur to calculate the total DNA index of the bone marrow.

*Bone marrow total DNA index* = *DNA OD value (A268)*/*femur weight (g).*

### 2.10 SIgA content in intestinal tissue

The contents of sIgA in the ileum and colon tissues were determined by ELISA assay kits. All experimental procedures were conducted in strict adherence to the manufacturer’s instructions provided with the reagents.

### 2.11 Oxidative stress indicators in intestinal tissue

Biochemical assay kits determined the contents of SOD and MDA in ileum and colon tissues. All experimental procedures were conducted in strict adherence to the manufacturer’s instructions provided with the reagents.

### 2.12 T lymphocyte subsets within IELs

The entire small intestine of the mouse was placed into Petri dish containing pre-cooled PBS, residual mesenteric fat tissue was removed, and Peyer’s patches were carefully excised. The intestine was longitudinally opened, and the intestinal contents were washed away. After cutting the small intestine into small pieces, it was digested with DTT and EDTA solution, the digested tissue was discarded, and the supernatant containing IELs was collected. This supernatant was sequentially passed through 200 and 400 mesh cell strainers to collect the filtrate, which, after centrifugation, was resuspended in 40% Percoll. The lymphocyte-containing 40% Percoll was gently layered on top of 70% Percoll for gradient separation. After the Percoll separation, the white cell layer between the two phases was extracted and centrifuged, the supernatant was discarded, and the cell pellet was resuspended with PBS solution to prepare a single-cell suspension. Under the microscope, cells were counted, and the cell concentration was adjusted to 10^5^-10^6^ cells/mL for subsequent experiments (Reißig et al., 2014).

0.2 mL of the lymphocyte suspension was taken, antibodies were added, and incubated in ice for 30 min. After incubation, the cells were washed twice to remove excess antibodies. The subpopulations of CD3⁺, CD4⁺, CD8⁺ T lymphocytes within the IELs were detected using flow cytometry.

### 2.13 Intestinal microbiota

Cecal contents from mice were collected under sterile conditions for 16S rDNA sequencing. DNA was extracted and amplified, and libraries were prepared for sequencing on the Illumina NovaSeq 6000.

### 2.14 Statistical methods

SPSS26.0 statistical software was used to analyze the data. *p <* 0.05 means a significant difference, *p <* 0.01 means a very significant difference, and *p >* 0.05 means no significant difference.

## 3 Results

### 3.1 The analysis results of active metabolites in HQGCD

#### 3.1.1 The content determination of calycosin and glycyrrhizic acid

**FIGURE 1:**
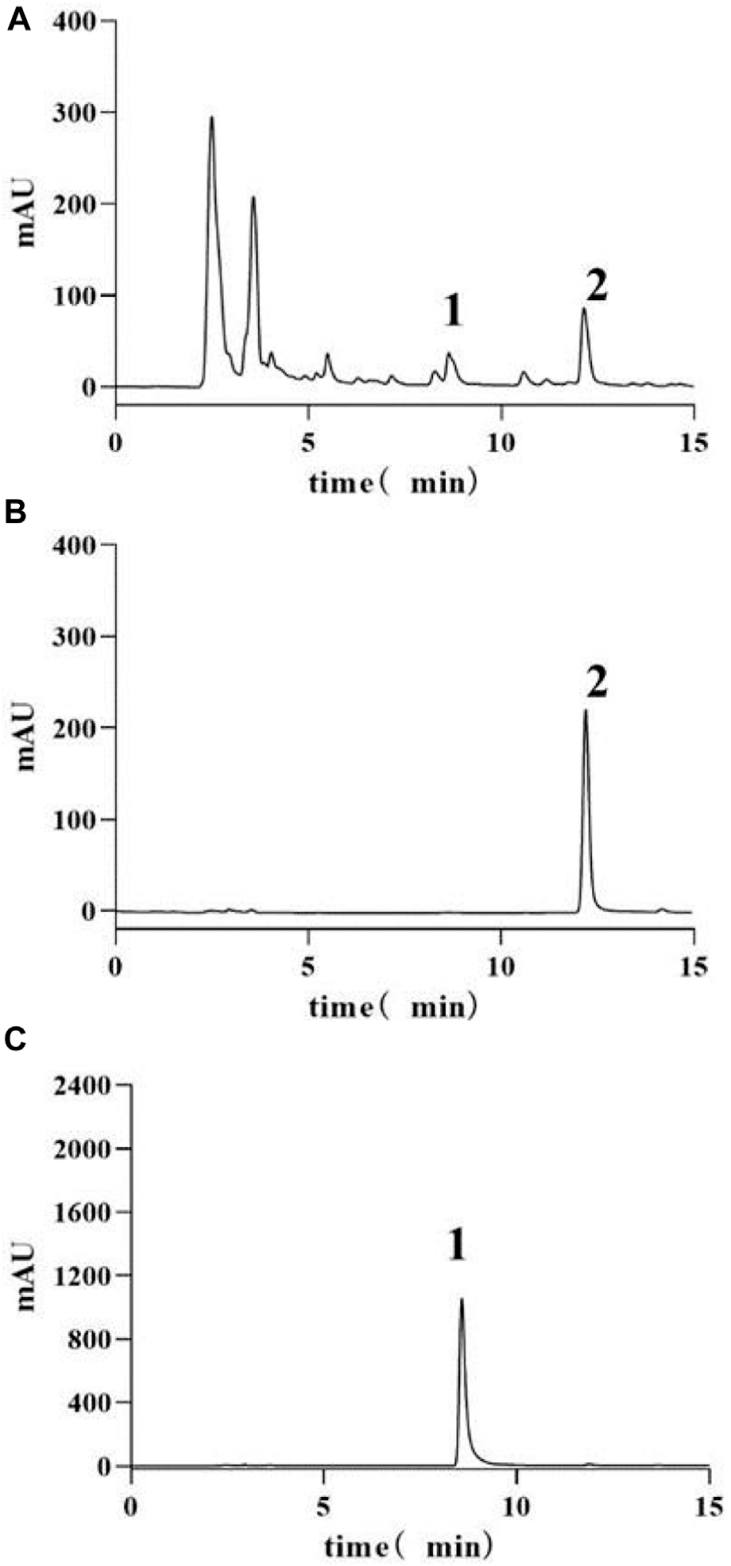
HPLC-UV chromatograms of samples and standards. Note: **(A)** HQGCD sample **(B)** Glycyrrhizic acid standard **(C)** Calycosin standard. 1. Peak representing calycosin 2. Peak representing glycyrrhizic acid.

The HPLC analysis of HQGCD revealed that the active metabolites, calycosin and glycyrrhizic acid, achieved baseline separation from other peaks, with retention times of 8.6 min and 12.1 min, respectively. Linear regression was conducted with peak area (x) as *x*-axis and content (y, μg/mL) as *y*-axis, yielding the regression equation for calycosin as: y = 0.01763x + 0.73931 (*R* ^2^ = 0.99997), with a detection range of 10.0152-10.6743 µg; and for glycyrrhizic acid as: y = 0.02616x - 0.14206 (*R* ^2^ = 0.99996), with a detection range of 30.2547-31.4982 µg (Supplementary Figure S1A,B). This method meets the requirements for HQGCD samples analysis. Based on the established standard curves for calycosin and glycyrrhizic acid, the contents of these metabolites in each group of HQGCD (10 mg/mL) were calculated to be 10.3825 μg/mL and 30.7960 μg/mL, respectively (Figure 1).

#### 3.1.2 HPLC chromatogram of three batches of HQGCD samples

**FIGURE 2:**
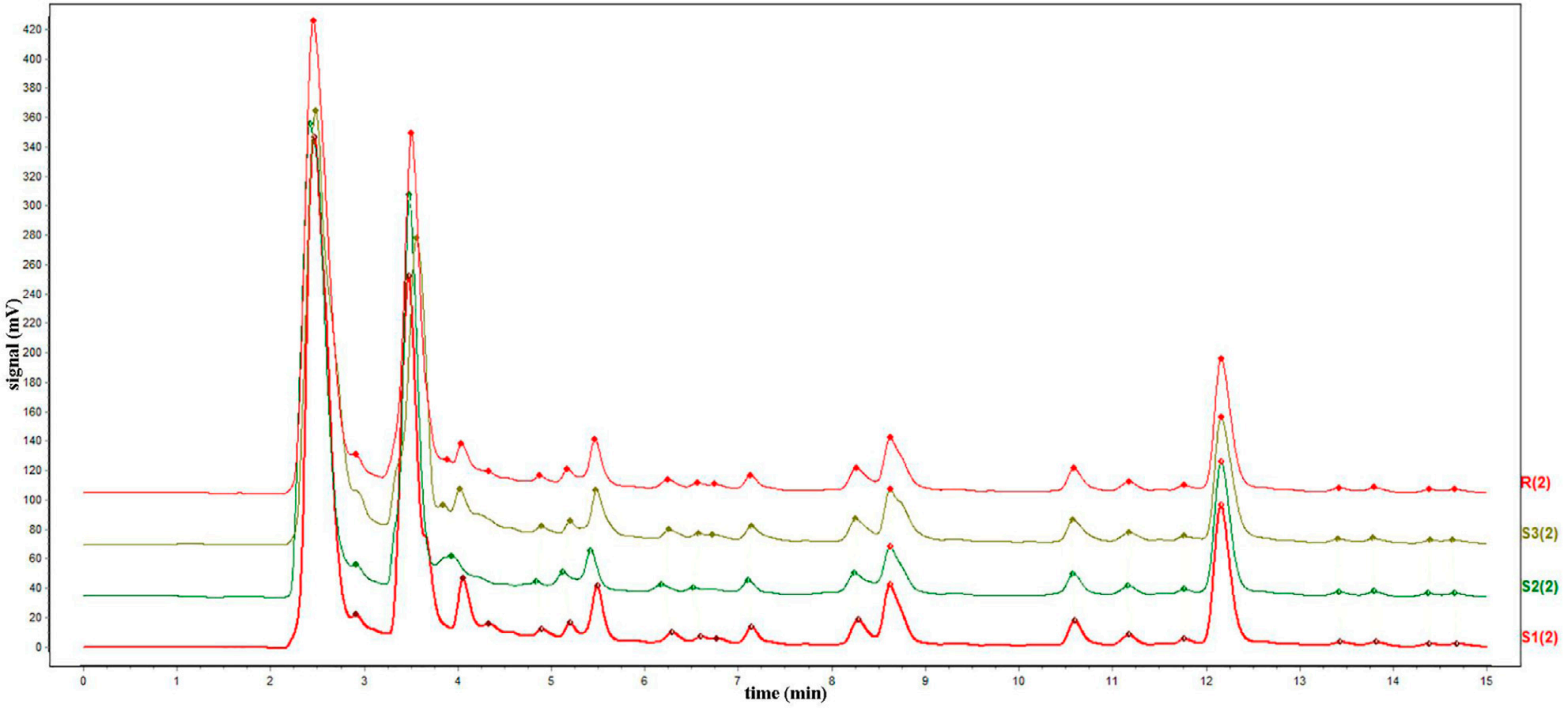
HPLC chromatogram of three batches HQGCD samples. Note: S1∼S3 denote the represent samples, and R denotes the reference peak.

The chromatograms of the three batches of HQGCD samples were highly overlapping (Figure 2), and the HPLC results of the samples were compared with each other, with a similarity of ≥0.998, which indicated this method had a good detection repeatability (Tables 1, 2).

**TABLE 1:** Similarity results.

	HQGCD 1	HQGCD 2	HQGCD 3	Control
HQGCD 1	1	0.994	0.996	0.998
HQGCD 2	0.994	1	0.997	0.999
HQGCD 3	0.996	0.997	1	0.999
Control	0.998	0.999	0.999	1

**TABLE 2:** Matching results.

Number	Retention time	HQGCD1	HQGCD 2	HQGCD 3	Control fingerprint	Retention time RSD (%)	Peak area RSD (%)	Number of matches
0	2.457	5607.855	5760.622	5520.259	5629.579	1.27	2.16	3
1	2.916	216.007	58.853	0	91.62	0.06	80.86	2
2	3.507	3396.465	2980.65	2745.384	3040.833	1.34	10.84	3
3	3.888	0	372.746	103.606	158.784	1.72	79.9	2
4	4.039	379.154	0	345.217	241.457	0.66	6.63	2
5	4.334	63.072	0	0	21.024	0	0	1
6	4.878	42.101	24.112	33.017	33.077	0.69	27.19	3
7	5.175	78.657	103.932	63.08	81.89	0.84	25.18	3
8	5.466	330.818	246.455	337.44	304.904	0.7	16.64	3
9	6.244	62.763	48.096	54.027	54.962	0.96	13.42	3
10	6.564	33.213	21.068	22.762	25.681	0.59	25.61	3
11	6.748	24.658	0	25.985	16.881	0.47	3.71	2
12	7.129	117.599	83.473	110.004	103.692	0.27	17.28	3
13	8.262	203.786	250.592	197.549	217.309	0.23	13.34	3
14	8.622	563.528	526.141	551.267	546.979	0	3.48	3
15	10.586	194.868	182.135	190.844	189.282	0.09	3.44	3
16	11.171	76.364	69.189	77.737	74.43	0.04	6.17	3
17	11.766	39.167	33.403	35.928	36.166	0.02	7.99	3
18	12.16	1209.489	1161.957	1176.494	1182.647	0	2.06	3
19	13.421	35.84	33.575	33.532	34.316	0.1	3.85	3
20	13.799	40.353	38.207	39.279	39.28	0.09	2.73	3
21	14.385	27.136	25.319	20.698	24.384	0.11	13.61	3
22	14.658	31.443	28.532	34.205	31.393	0.17	9.04	3

### 3.2 General behavioral observations

**FIGURE 3:**
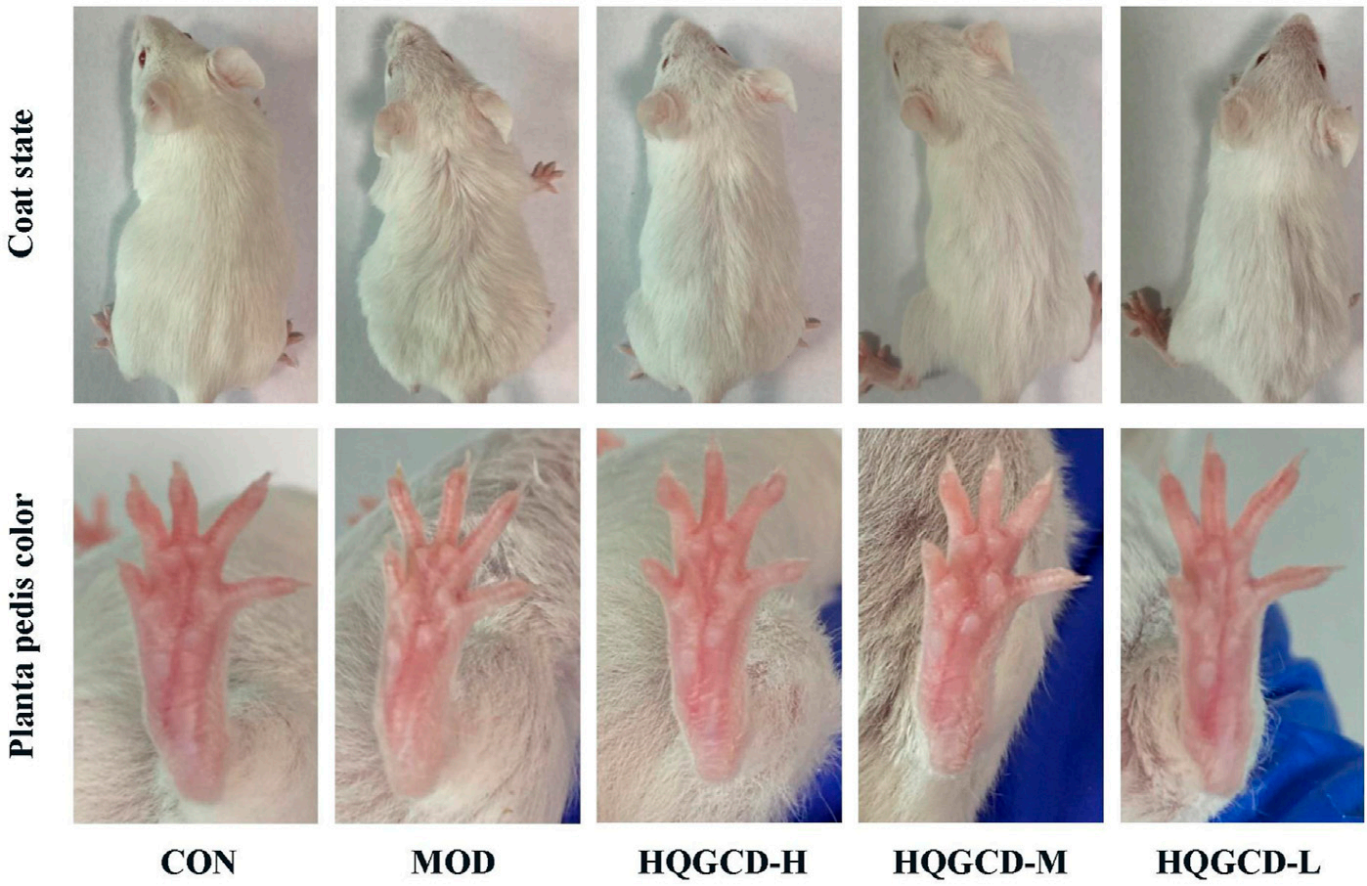
Effect of HQGCD on coat state and planta pedis color in mice.

Unlike CON, mice in MOD exhibited lethargy, laziness and tiredness, reduced reaction ability and activity, dry and dull fur, and lighter planta pedis color, indicating successful modeling. After treatment with HQGCD, all three dose groups showed improvement (Figure 3).

### 3.3 Body weight and feed intake

**FIGURE 4:**
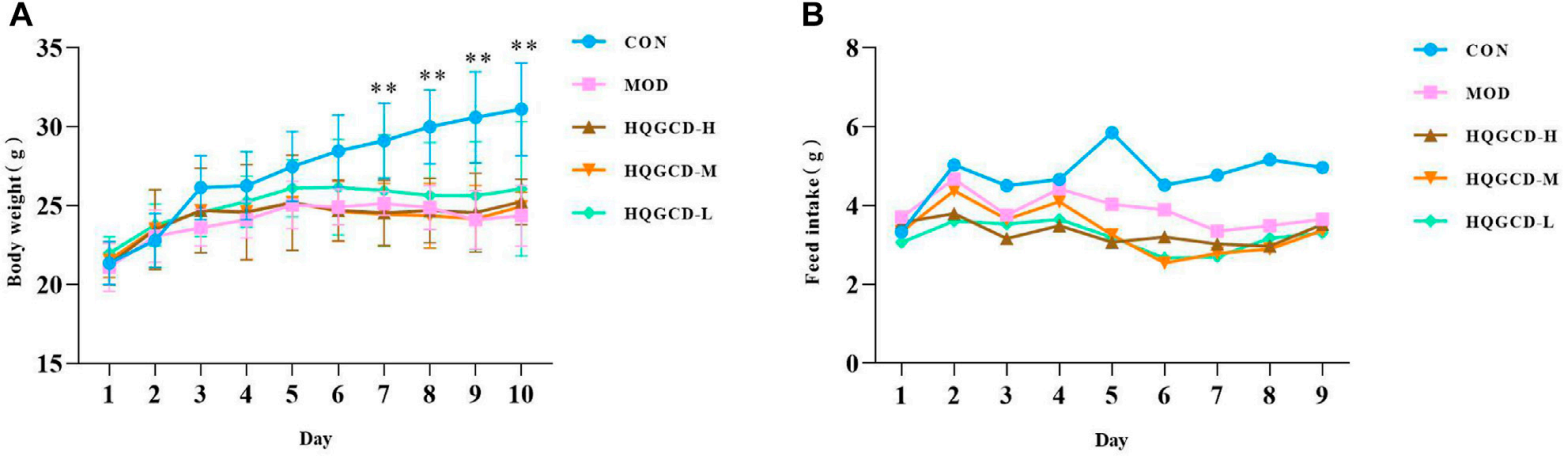
Effect of HQGCD on body weight and feed intake in mice. Note: **(A)** body weight **(B)** feed intake. Data were presented as mean ± SD [A n = 8]^. **^ *p <* 0.01, ^*^ *p <* 0.05 vs MOD; ^##^ *p <* 0.01, ^#^ *p <* 0.05 vs CON.

Body weight gain was slower in both MOD and three dose groups than CON (Figure 4A), and it showed a very significant difference at day 7 (*p <* 0.01). However, no significant difference was observed when comparing the growth of the three dose groups to MOD (*p >* 0.05). Compared to CON, the feed intake decreased in both MOD and three dose groups (Figure 4B).

### 3.4 Ileal and colonic tissue sections

**FIGURE 5:**
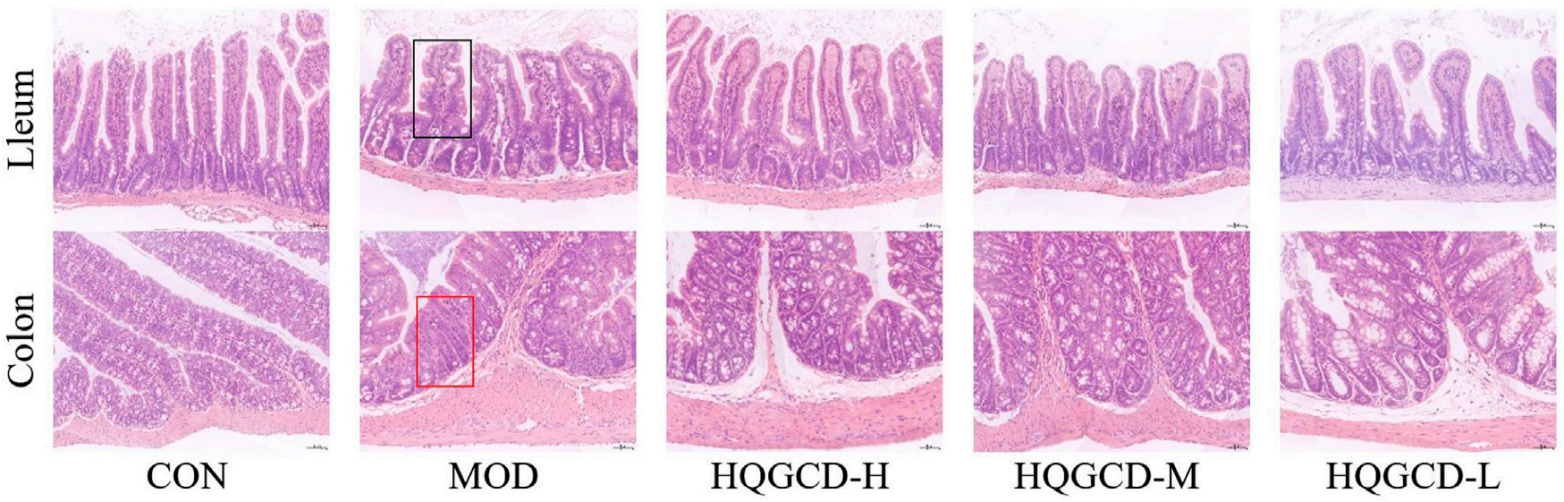
Effect of HQGCD on intestinal morphology in mice (HE × 200). Note: Atrophic and deformed ileal villi are seen in the black rectangle; The colon with reduced colorectal glands is shown in the red rectangle.

The ileal mucosa of CON was intact, with neatly arranged villi and intestinal epithelial cells that were morphologically distinct, tightly arranged, and uniform in size, showing no obvious pathological changes. In contrast, the ileum of MOD exhibited deformation and atrophy, with a reduction in the length of the intestinal villi. The ileal villi of three dose groups showed recovery compared to MOD. The colonic mucosa of CON was intact without obvious pathological changes. In contrast, the colonic mucosal layer of MOD was infiltrated with inflammatory cells, and the number of colorectal glands in the colon were reduced. The colon of three dose groups recovered relative to MOD (Figure 5).

### 3.5 Blood routine index

**FIGURE 6:**
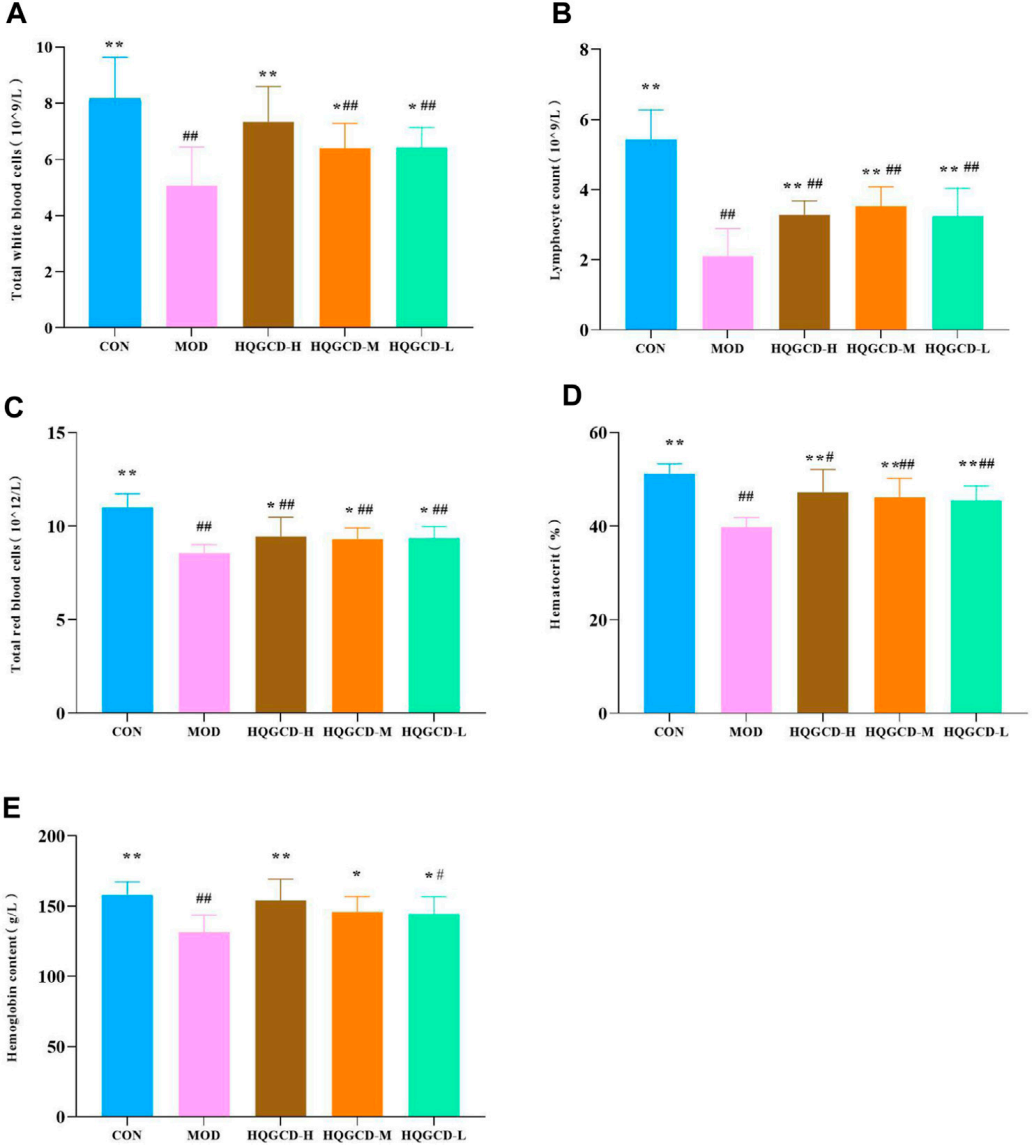
Effect of HQGCD on routine blood index in mice. Note: **(A)** Total white blood cells **(B)** Lymphocytes count **(C)** Total red blood cells **(D)** Hematocrit **(E)** Hemoglobin content. Data were presented as mean ± SD [A-E n = 8]. ^**^ *p <* 0.01, ^*^ *p <* 0.05 vs MOD; ^##^ *p <* 0.01, ^#^ *p <* 0.05 vs CON.

Relative to CON, the total white blood cells, lymphocyte count, total red blood cells, hematocrit and hemoglobin content very significantly decreased in MOD (*p <* 0.01). Relative to MOD, these parameters were significantly increased in three dose groups (*p <* 0.05), HQGCD-H of the total white blood cell count and three dose groups of hematocrit were very significantly increased (*p <* 0.01) (Figures 6A–E).

### 3.6 Bone marrow total DNA index

**FIGURE 7:**
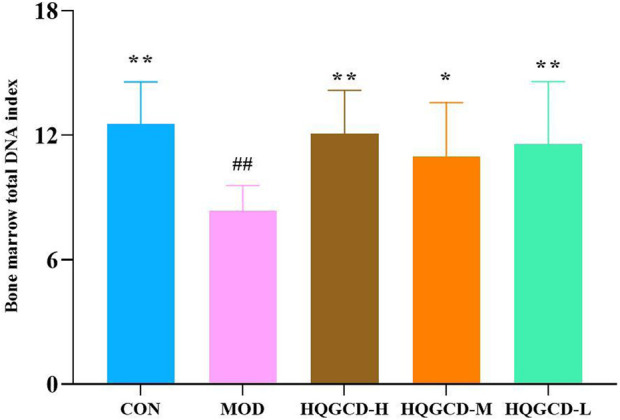
Effect of HQGCD on mouse bone marrow total DNA index. Note: Data were presented as mean ± SD [n = 8]. ^**^ *p <* 0.01, ^*^ *p <* 0.05 vs MOD; ^##^ *p <* 0.01, ^#^ *p <* 0.05 vs CON.

Compared with the bone marrow total DNA index of CON, MOD was very significantly reduced (*p <* 0.01). Compared with MOD, HQGCD-H and HQGCD-L were evry significantly increased (*p* <0.01). HQGCD-M showed a significantly increased (*p <* 0.05) (Figure 7).

### 3.7 SIgA level in intestinal tissues

**FIGURE 8:**
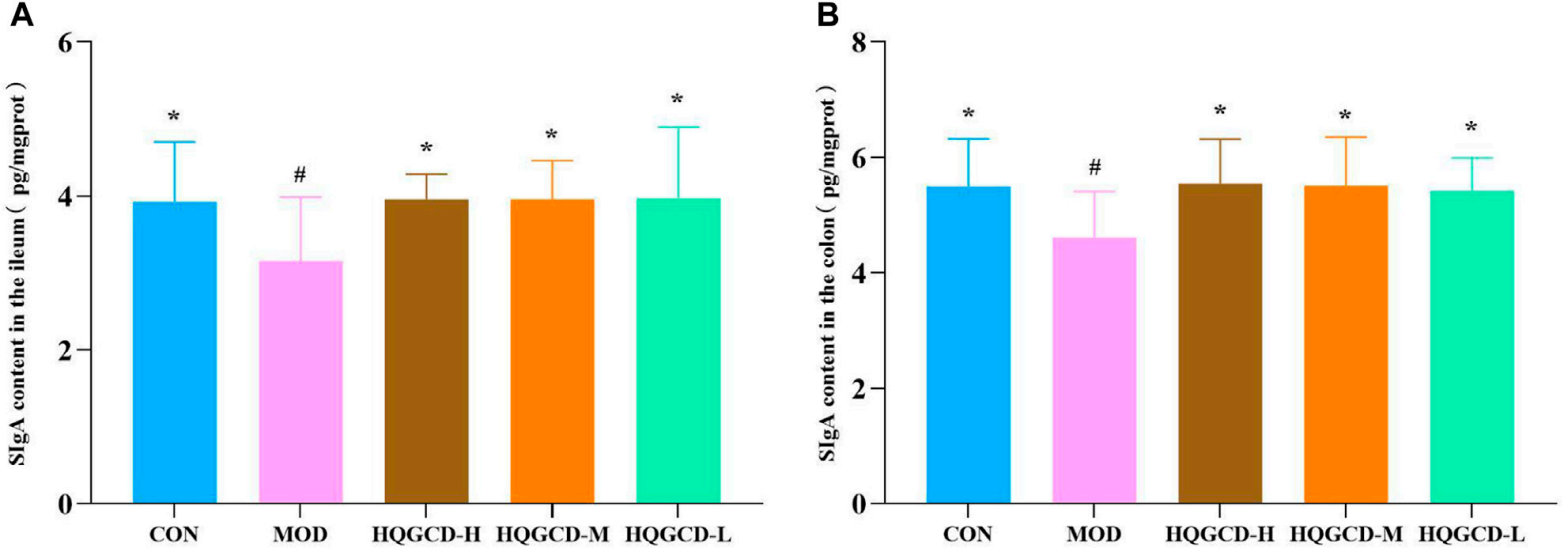
Effect of HQGCD on sIgA in mouse intestinal tissue. Note: **(A)** sIgA content in the ileum **(B)** sIgA content in the colon. Data were presented as mean ± SD [A-B n = 8]. ^**^ *p <* 0.01, ^*^ *p <* 0.05 vs MOD; ^##^ *p <* 0.01, ^#^ *p <* 0.05 vs CON.

In comparison with the level of sIgA in the ileum and colon of CON, MOD was significantly decreased (*p <* 0.05); In comparison with the level of sIgA in the ileum and colon of MOD, three dose groups showed significantly increased (*p <* 0.05) (Figures 8A, B).

### 3.8 MDA and SOD level in intestinal tissue

**FIGURE 9:**
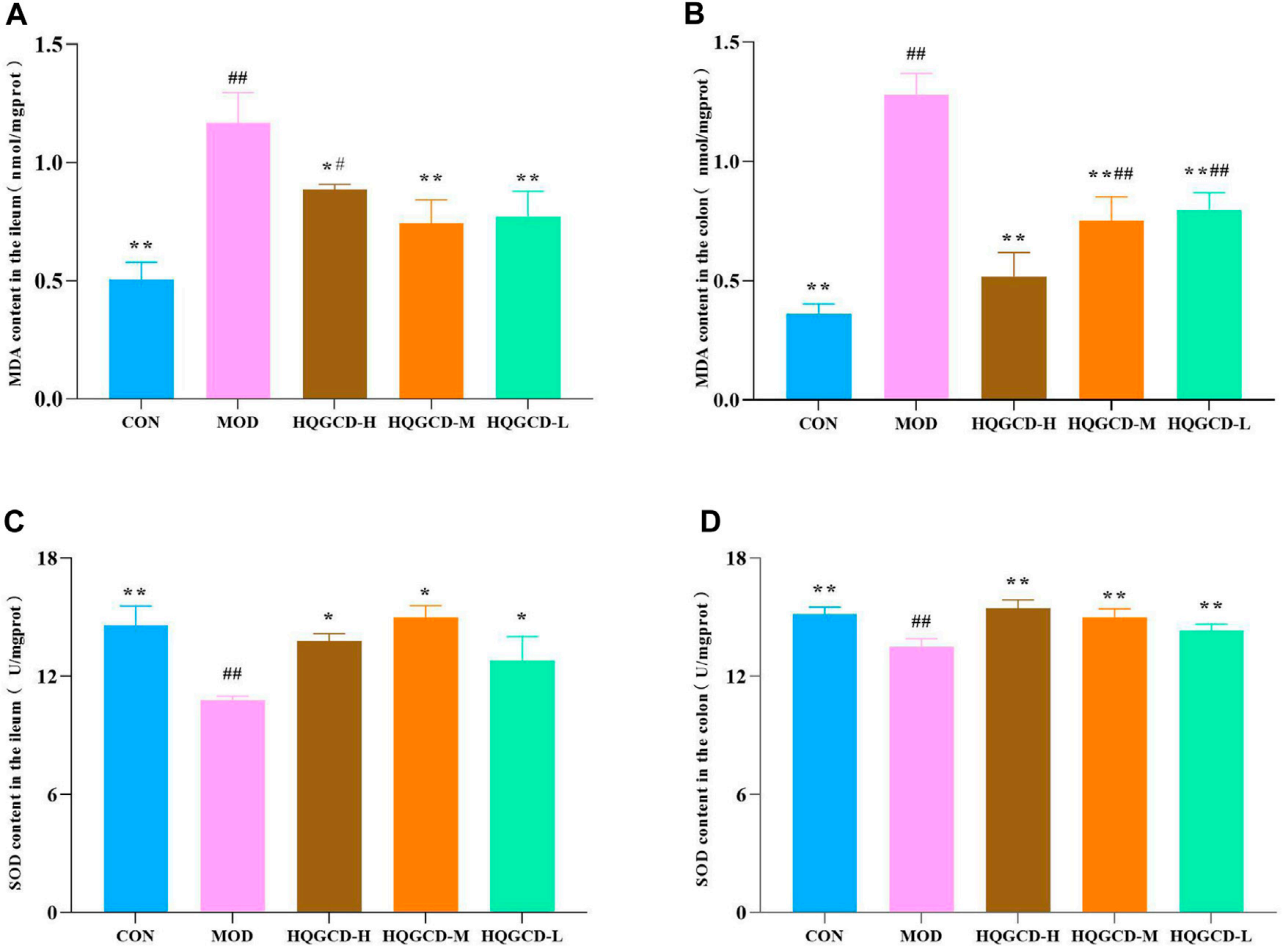
Effect of HQGCD on MDA and SOD in mouse intestinal tissue. Note: **(A)** MDA content in the ileum **(B)** MDA content in the colon **(C)** SOD content in the ileum **(D)** SOD content in the colon. Data were presented as mean ± SD [A-E n = 8]. ^**^ *p <* 0.01, ^*^ *p <* 0.05 vs MOD; ^##^ *p <* 0.01, ^#^ *p <* 0.05 vs CON.

As opposed to the MDA level in the ileum and colon tissues of CON, the MOD level was very significantly increased (*p <* 0.01). As opposed to MOD, the MDA level in the ileum tissue of HQGCD-H was significantly decreased (*p <* 0.05), while in the remaining five dose groups, MDA level in both the ileum and colon tissues was very significantly decreased (*p <* 0.01) (Figures 9A, B).

Relative to the SOD level in the ileum and colon tissues of CON, the level in MOD was very significantly decreased (*p <* 0.01). Relative to MOD, SOD level in the colon tissues of three dose groups was very significantly increased (*p <* 0.01). In the ileum tissues of three dose groups, the increase was significant (*p <* 0.05) (Figures 9C, D).

### 3.9 T lymphocyte subsets within IELs

**FIGURE 10:**
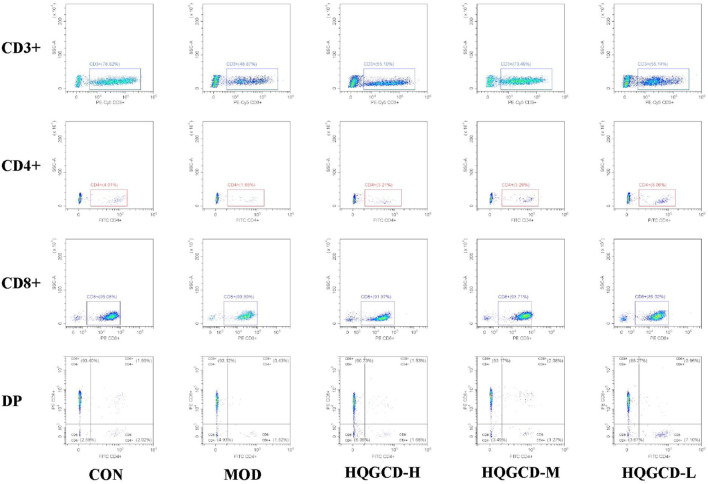
Effect of HQGCD on T lymphocyte subsets within IELs.

CD3⁺ T Lymphocytes: in contrast with the CD3⁺ T lymphocyte level in LELs of MOD, both CON and three dose groups exhibited an upward trend, although this trend was not statistically significant (*p >* 0.05); CD4⁺ T Lymphocytes: relative to the CD4⁺ T lymphocytes in IELs of MOD, three dose groups showed an upward trend, but this trend was not significant (*p >* 0.05). CD8⁺ T Lymphocytes: compared to the CD8⁺ T lymphocytes in IELs of MOD, both CON and three dose groups displayed a downward trend, which was not significant (*p >* 0.05). DP T Lymphocytes: in comparison with the DP IELs of CON, MOD and HQGCD-M showed significant differences (*p <* 0.05), whereas HQGCD-H and HQGCD-M exhibited an upward trend that was not statistically significant (*p >* 0.05). When compared to MOD, three dose groups showed an upward trend, but this trend was insignificant (*p >* 0.05). No significant differences were observed in the level of DP IELs among the three dose groups (*p >* 0.05) (Figure 10; Table 3).

**TABLE 3:** Comparison of changes in different types of T lymphocyte subsets in mice IELs (%).

Items	CD3⁺	CD4⁺	CD8⁺	DP
CON	62.63 ± 15.95	6.72 ± 2.95	92.16 ± 3.06	3.03 ± 1.39^*^
MOD	50.66 ± 10.04	2.58 ± 0.86	92.64 ± 2.15	0.39 ± 0.05^#^
HQGCD-H	59.23 ± 8.66	5.79 ± 4.75	91.50 ± 4.14	1.85 ± 1.00
HQGCD-M	63.35 ± 19.13	4.02 ± 2.22	91.37 ± 3.06	1.28 ± 0.71
HQGCD-L	59.88 ± 18.43	4.40 ± 3.49	92.22 ± 4.05	1.05 ± 0.82^*^

Data were presented as mean ± SD [*n* = 3].^**^ *p <* 0.01, ^*^ *p <* 0.05 vs MOD;^##^ *p <* 0.01, ^#^ *p <* 0.05 vs CON.

### 3.10 Sequencing results of intestinal microbiota

**FIGURE 11:**
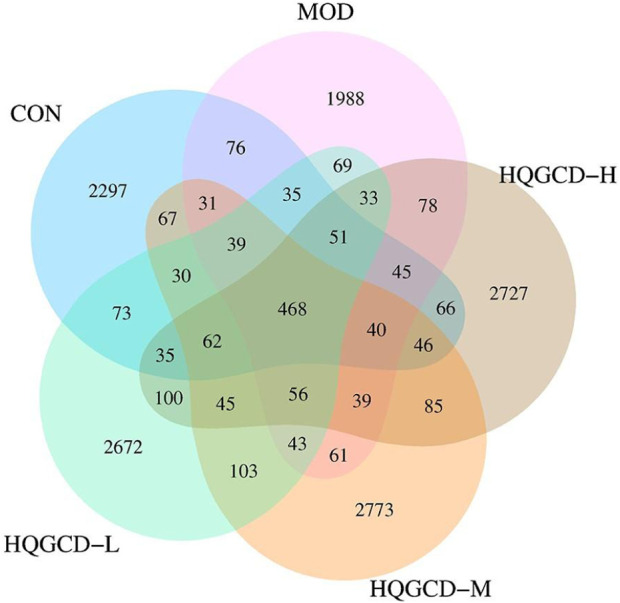
Venn diagram of ASV/OUT of microbiota.

In this experiment, 40 samples were analyzed, yielding 2,333,722 sequences with an average of 58,343 reads per sample. To explore the commonalities and differences among various groups, a Venn diagram based on the ASV/OTU abundance was employed for community composition analysis. The analysis identified 468 species shared across CON, MOD, HQGCD-H, HQGCD-M, and HQGCD-L. Additionally, unique endemic species were identified for each group: 2,297 for CON, 1,988 for MOD, 2,727 for HQGCD-H, 2,773 for HQGCD-M, and 2,672 for HQGCD-L (Figure 11).

### 3.11 Alpha diversity analysis

**FIGURE 12:**
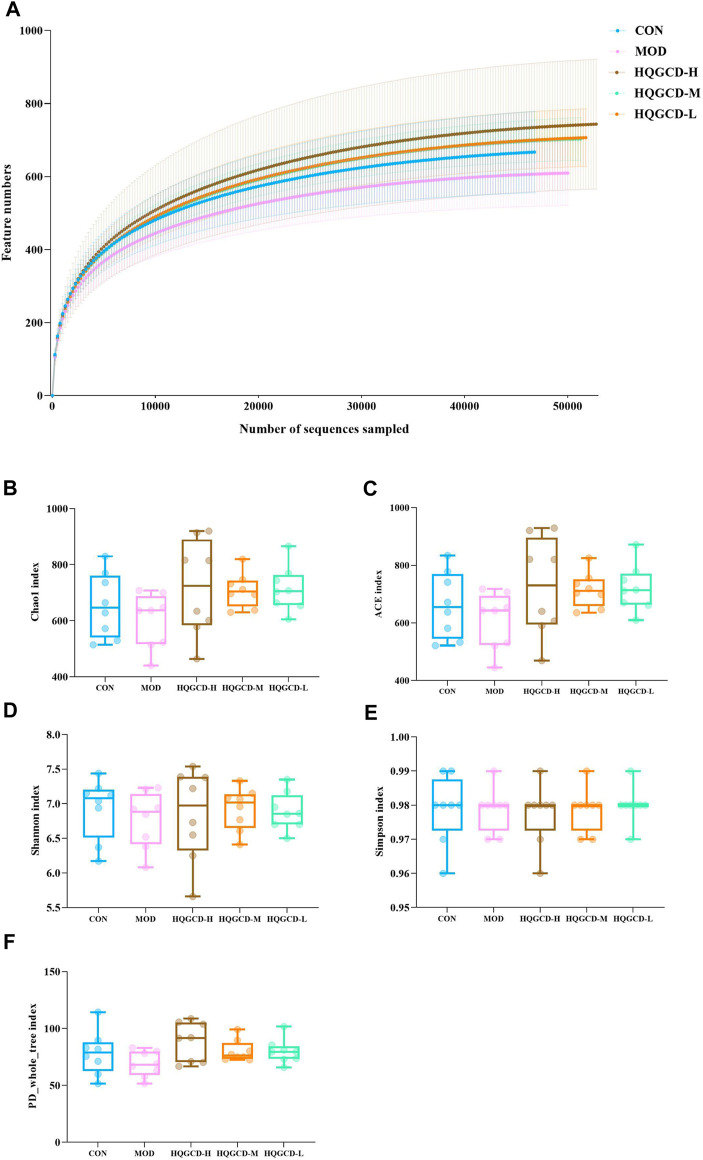
Alpha diversity analysis. Note: **(A)** Sparse curves of microbiota species **(B)** Chao1 index **(C)** Ace index **(D)** Shannon index **(E)** Simpson index **(F)** PD_whole_tree index.

Dilution curves were utilized to determine if the volume of sequencing data was adequate to represent the species diversity within the samples, indirectly indicating the samples’ species richness. In this experiment, the dilution curves for all five groups plateaued towards the end, signifying that the sequencing depth was sufficient to encompass the species composition of these samples (Figure 12A). Alpha diversity assesses the species richness and diversity within individual samples of a group, utilizing multiple indicators, including Chao1, Ace, Shannon, Simpson, and PD_whole_tree. The results indicated that the coverage for each group exceeded 0.99. When measured against CON, the Chao1, Ace, Shannon, PD_whole_tree and Simpson indices of MOD showed a downward trend, though this trend was insignificant (*p >* 0.05). In contrast, three dose groups exhibited an upward trend compared to MOD, but the increase was not significant (*p >* 0.05) (Figures 12B–F).

### 3.12 Beta diversity analysis of microbiota

**FIGURE 13:**
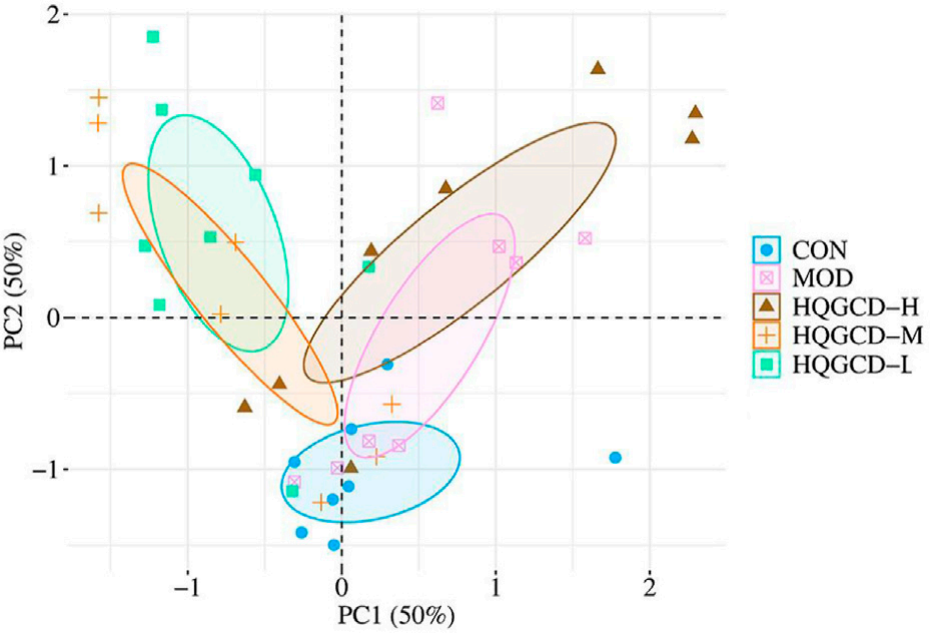
PCA analysis of microbiota.

Principal component analysis (PCA) reflects the differences and distances among samples. It was utilized to examine the Beta diversity of the samples within a 68% confidence interval. The analysis revealed a trend where the community composition of CON tended to separate from the other four groups (Figure 13).

### 3.13 Composition of the top 10 biological species at the phylum and genus level of mouse intestinal microbiota

**FIGURE 14:**
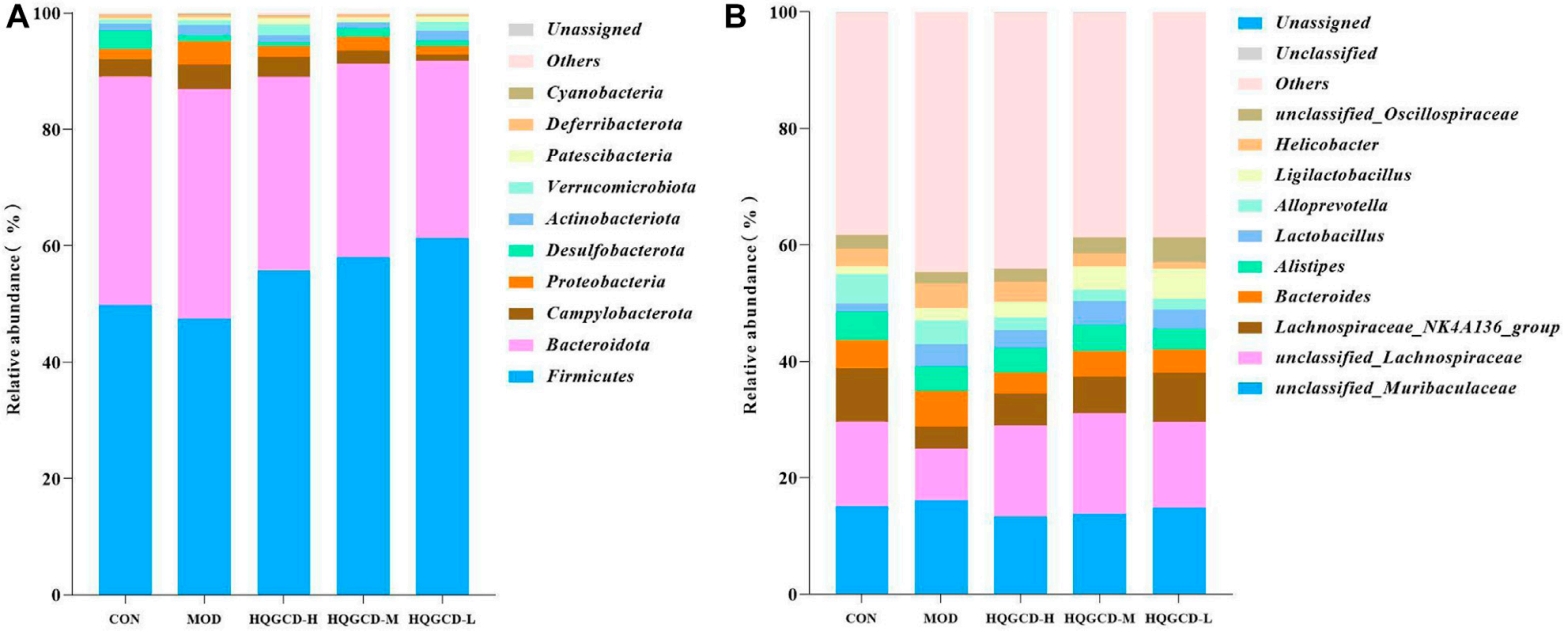
Note: **(A)** Relative abundance of microflora at phylum level. **(B)** Relative abundance of microflora at genus level.

At the phylum level, *Firmicutes*, *Bacteroidetes*, *Campylobacterota*, and *Proteobacteria* were predominant in the fecal microbiota. As opposed to CON, the relative abundance of *Firmicutes* in MOD decreased, while it increased in three dose groups. When comparing three dose groups to MOD, the relative abundance of *Firmicutes* increased. Conversely, the relative abundance of *Bacteroidetes* in MOD increased compared with CON, but decreased in three dose groups. The relative abundance of *Bacteroidetes* in three dose groups also decreased in relation to MOD. The relative abundance of *Campylobacterota* increased in MOD compared with CON. Specifically, it increased in HQGCD-H but decreased in HQGCD-M and HQGCD-L. Relative to MOD, the relative abundance of *Campylobacterota* in three dose groups decreased. Lastly, the relative abundance of *Proteobacteria* in MOD increased compared with CON, with an increase in HQGCD-H and HQGCD-M, but a decrease in HQGCD-L. The relative abundance of *Proteobacteria*in three dose groups decreased in relation to MOD (Figure 14A).

At the genus level, *unclassified_Muribaculaceae*, *unclassified_Lachnospiraceae*, *Lachnospiraceae_NK4A136_group*, *Bacteroides*, *Alistipes*, and *Lactobacillus* were the predominant fecal microbiota. Compared with CON, the relative abundance of *unclassified_Muribaculaceae* in MOD was increased, and three dose groups were decreased. Compared with MOD, the relative abundance of *unclassified_Muribaculaceae* in three dose groups was decreased. In contrast with CON, the relative abundance of *unclassified_Lachnospiraceae* in MOD was decreased, and three dose groups were increased. In contrast with MOD, the relative abundance of *unclassified_Lachnospiraceae* in three dose groups was increased. As opposed to CON, the relative abundance of *Lachnospiraceae_NK4A136_group* in MOD and three dose groups was decreased. As opposed to MOD, the relative abundance of *Lachnospiraceae_NK4A136_group* in three dose groups was increased. Relative to CON, the relative abundance of *Bacteroides* in MOD was increased, and three dose groups was decreased. Relative to MOD, the relative abundance of *Bacteroides* in three dose groups was decreased. Compared with CON, the relative abundance of *Alistipes* in MOD was decreased, three dose groups was decreased. Compared with MOD, the relative abundance of *Alistipes* in HQGCD-H and HQGCD-M was increased, and HQGCD-L was decreased. In comparison with CON, the relative abundance of *Lactobacillus* in MOD and three dose groups increased. In comparison with MOD, the relative abundance of *Lactobacillus* in HQGCD-H and HQGCD-M was increased, and HQGCD-L was decreased (Figure 14B).

### 3.14 LEfSe analysis of samples between groups

**FIGURE 15:**
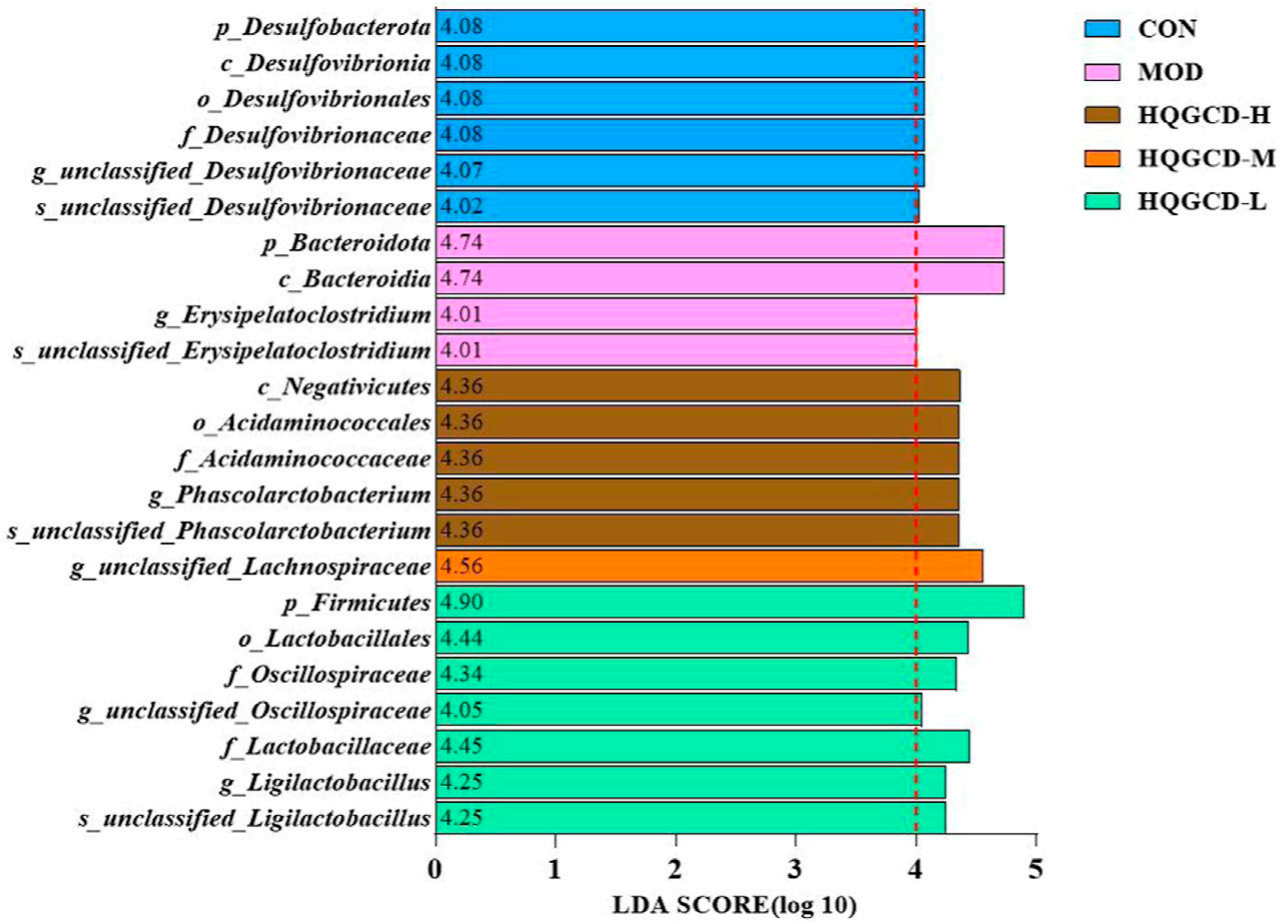
Distribution of LDA values in microbiota.

LEfSe analysis can be used to compare two or more groups to identify species with significant differences in abundance between groups. LEfSe analysis of species with significant differences between groups showed that 23 microbial groups with LDA value greater than four in the five groups played an important role in the differences. CON enrichment *f_Lachnospiraceae_NK4A136_group*, *p_Desulfobacterota*, *c_Desulfovibrionia*, *o_Desulfovibrionales*, *g_unclassifie_Desulfovibrionaceae*, *s_unclassifie_Desulfovibrionaceae*; MOD enrichment *p_Bacteroidota*, *c_Bacteroidia*, *s_unclassified_Erysipelatoclostridium*, *g_Erysipelatoclostridium*; HQGCD-H enrichment *c_Negativicutes*, *f_Acidaminococcaceae*, *o_Acidaminococcales*, *g_Phascolarctobacterium*, *s_unclassified_Phascolarctobacterium*; HQGCD-M enrichment *g_unclassified_Lachnospiraceae*; HQGCD-L enrichment *p_Firmicutes*, *f_Lactobacillaceae*, *o_Lactobacillales*, *f_Oscillospiraceae*, *s_unclassified_Ligilactobacillus*, *g_Ligilactobacillus*, *g_unclassified_Oscillospiraceae* (Figure 15).

## 4 Discussion

In animals, the underlying cause of many diseases is low or poor immunity. Traditional Chinese medicine believe that “as long as the “Qi” is present within the body, pathogens cannot prevail”. Here, “Qi” symbolizes the vitality of the body’s functions, encompassing its capability to adapt to external conditions, resist diseases, and recover from illness. A deficiency in “Qi” can result in an imbalance of “Yin” and “Yang” within the body, disruption in the circulation of “Qi” and blood among the viscera, and a consequent susceptibility to diseases due to a weakened constitution. While chemical drugs offer certain benefits in enhancing immune function, they are often associated with significant side effects and safety concerns (Hashimoto et al., 2023). In contrast, traditional Chinese medicine’s immunomodulatory approach is comprehensive, characterized by minimal side effects and toxicity (Nasri et al., 2014). The therapeutic strategy in traditional Chinese medicine aims to “nourish the body and strengthen its foundation” by enhancing or regulating bodily functions, thereby improving the body’s disease resistance and fulfilling the objectives of disease prevention and treatment (Peng et al., 2022).

HQGCD, derived from the Qing Dynasty medical book “*Yi Lin Gai Cuo*”, is primarily treated for urinary tract diseases. However, *A. membranaceus* and *G. uralensis* in the prescription contain polysaccharides, flavonoids, and saponins (Deng et al., 2023), so it is speculated that HQGCD can improve immunity, which has been confirmed in this experiment. Additionally, this study quantified the metabolites of calycosin in *A. membranaceus* and glycyrrhizic acid in *G. uralensis* using HPLC, conducted calibration and similarity assessment of the common peaks for calycosin and glycyrrhizic acid to achieve better quality control of the HQGC

Cy is a highly effective chemotherapeutic drug and immunosuppressant known for side effects such as severe bone marrow suppression, destruction of the body’s normal immune cells, and gastrointestinal adverse reactions (Fraiser et al., 1991). In this experiment, a dose of 80 mg/kg Cy was administered to establish the model, and the immunosuppression induced by Cy is systemic; therefore, the intestine also undergoes immunosuppression.

Three days post-modeling, the mice exhibited symptoms of depression, reduced activity, decreased feed intake, slow body weight gain, disheveled fur, and whitening of the planta pedis, confirming the model’s success. After HQGCD intervention, there was no significant increase in the mice’s feed intake, possibly due to the high polysaccharide content in HQGCD providing some energy. Furthermore, the observed weight gain was not significant, indicating that HQGCD does not substantially influence the weight loss induced by Cy, aligning with findings from Yang et al. (Zhengli et al., 2023).

Cy nonspecifically disrupts and inhibits DNA synthesis in bone marrow hematopoietic cells, extends the cell cycle, diminishes the production of mature blood cells, and lowers the count of nucleated cells. This leads to anemia and immunosuppression (Yu et al., 2018), as well as a reduction in bone marrow total DNA content. The blood routine can detect the number of white blood cells, red blood cells and platelets in the blood, thus indicating the toxic impact of Cy on mice blood cells, it is also regarded as one of standards for successfully establishing an immunosuppression model using Cy. Since the bone marrow is the primary hematopoietic organ, the bone marrow total DNA content indicates the extent of bone marrow cell damage and recovery. This study found that Cy administration decreased blood cell counts and bone marrow total DNA content. Following HQGCD intervention, improvements were observed in the blood routine content and total bone marrow DNA content.

The intestine is not only crucial for the digestion and absorption of food but also serves as the body’s largest immune organ. Its lumen is lined with epithelial cells, which are interconnected by tight junction proteins and shielded by a layer of protective mucus secreted by glandular cells within the epithelium. The epithelial layer and mucus layers form a barrier structure that can physically defend against most pathogens and act as the first line of defense. The intestinal structure can be clearly observed in tissue staining sections, and the ileum and colon are the most developed parts of the mucosal immunity of the small intestine and large intestine, respectively, so the ileum and colon were taken for staining section observation in this experiment. After Cy modeling, the ileum villi were deformed and atrophied, the length of intestinal villi was shortened, and the number of large intestinal glands in the colon was reduced, but the degree of damage was not deep, which may be related to the dose of Cy. That can be restored to a certain extent after HQGCD intervention.

IgA, the predominant immunoglobulin in the intestine, plays a crucial role in mucosal immunity and intestinal defense. It can recognize lipopolysaccharides, capsular polysaccharides and flagella on the surface of certain bacteria, form sIgA-coated bacteria, prevent pathogens from contacting intestinal epithelial cells, and thus protect the intestine (Pietrzak et al., 2020; Ding et al., 2021). Furthermore, sIgA can neutralize harmful enzymes and enterotoxins present in the intestine (Macpherson et al., 2001) and contributes to maintaining the dynamic equilibrium of the mucosal surface’s internal environment (Kato et al., 2014). A higher concentration of sIgA on the mucosal surface correlates with increased resistance to pathogens. Administration of Cy has been shown to decrease sIgA level in the intestinal mucosa, whereas HQGCD treatment can elevate sIgA level in mice’s ileum and colon tissues.

Oxidative stress and immunity are interrelated. Oxidative stress can lead to autoimmune diseases, while the immune system can activate and regulate the oxidative system. Oxidative stress is a series of adaptive responses caused by the imbalance between reactive oxygen species and the antioxidant system. MDA and SOD are oxidative and antioxidant indicators, respectively. MDA, which can induce cross-linking polymerization of proteins, nucleic acids, and other macromolecules, possesses cytotoxic properties and is an end product of free radical-induced lipid peroxidation in organisms (Xinchang et al., 2024). Conversely, SOD plays a critical role in eliminating oxygen free radicals, protecting tissues from free radical and peroxide damage. A reduction in SOD activity is associated with the extent of tissue damage (Schieber and Chandel, 2014). The two have opposite physiological effects. When Cy was applied to the intestine, the metabolism of intestinal cells was disordered, the level of MDA was increased, and the level of SOD was decreased. Intervention with HQGCD, however, results in decreased MDA level and increased SOD level, indicating a mitigative effect on oxidative stress.

The intestine serves as a vital digestive organ and the largest immune organ within the animal body. Intestinal mucosal epithelial cells are a monolayer of cells located in the inner part of the intestinal lumen, which is in direct contact with the external environment and can prevent pathogenic microorganisms and other harmful compounds from entering the body (Blikslager et al., 2007). The intestinal immune system comprises intestinal-associated lymphoid tissue and diffuse lymphoid tissue, with intestinal-associated lymphoid tissue consisting of IELs and lamina propria lymphocytes. The diffuse lymphoid tissue is a primary site for intestinal immunity (Pérez-Bosque et al., 2008). IELs are distributed throughout the epithelium of all mucosal and barrier sites, predominantly in the proximal small intestine, and decrease toward the colon. Most IELs are T lymphocytes of the small intestine. Dysregulation of IELs often leads to compromised mucosal barrier integrity, increased susceptibility to infections, and chronic inflammation (Hoytema van Konijnenburg and Mucida, 2017). CD3⁺ T lymphocytes are mature T cells whose level can reflect the total number of T lymphocytes. CD3⁺ T lymphocytes are an important indicator for evaluating the immune function of the body. According to their surface markers, CD3⁺ T lymphocytes can be divided into CD4⁺ and CD8⁺ T lymphocytes, with CD4⁺ cells functioning as T helper cells that regulate the differentiation of other immune cells and orchestrate the immune response. Conversely, CD8⁺ T lymphocytes belong to suppressor T cells, which can directly kill cells and play a negative regulatory role in immune response (Liu, 2023). In this study, compared with CON, the number of intraepithelial CD3⁺ and CD4⁺ T lymphocytes were decreased in MOD, the number of intraepithelial CD8⁺ T lymphocytes were increased in MOD. This study revealed that, compared to CON, MOD exhibited a decrease in the number of intraepithelial CD3⁺ and CD4⁺ T lymphocytes, while the number of CD8⁺ T lymphocytes increased. Following HQGCD treatment, there was an increase in the numbers of CD3⁺ and CD4⁺ T lymphocytes and a decrease in CD8⁺ T lymphocytes of three dose groups compared to MOD. These findings suggest that HQGCD effectively mitigates intestinal immunosuppression in mice.

DP T lymphocytes are commonly found in immature T lymphocytes, and mature T lymphocytes that truly co-express CD4⁺ and CD8⁺ are rare. In humans, DP T lymphocytes are usually present in low numbers in peripheral blood but can increase in lymphoma cases, certain viral infections, autoimmune diseases, and chronic inflammation (Fowlkes and Pardoll, 1989). Notably, DP T lymphocytes are observed in the peripheral blood and secondary lymphoid organs of pigs, monkeys, and chickens, which assist in immune function and increase with age (Zuckermann, 1999). However, there are few studies on mouse DP IELs, and some scholars have suggested that DP IELs in mice may be fully differentiated functional cells. The co-expression of CD4⁺ and CD8⁺ labeled IELs may contribute to the high-affinity attachment of effector IELs to epithelial tissues after TCR recognition of foreign antigens. However, this claim has not been proven (Mosley et al., 1990). Mouse DP IELs are known to express Th2-type cytokines, such as IL-5 and IL-6, and possess the capacity to mitigate Th1-induced intestinal inflammation. DP IELs can also produce IL-10, offering protection against Th1-induced intestinal inflammation, akin to the regulatory role of CD4⁺, CD25⁺ regulatory T cells, and B cells in an IL-10-dependent manner. The mechanism by which IL-10 from the small intestine provides protection to the large intestine is yet to be fully elucidated (Asseman et al., 1999; Das et al., 2003). In this experiment, compared with MOD, the number of DP IELs in CON and three dose groups after treatment were increased, proving that HQGCD could increase the number of DP IELs in mice to improve intestinal immunity. The difference is that the animals used in this experiment were KM mice, and no scholars have studied DP IELs in this breed of mice before.

The intestinal microbiota is intricately connected to the host’s health, forming a complex ecosystem that plays a vital role in nutrition, immunity, and disease resistance. However, this balance can be easily disrupted by various pathogenic factors, leading to alterations in the composition and function of the intestinal microbiota, which may contribute to a range of health issues (Adak and Khan, 2019). The results of intestinal microbiota sequencing showed that Cy reduced the diversity of the microbiota in the mice’s cecum, while HQGCD increased it. The Chao1 and Ace indices were utilized to estimate species richness, while the Shannon and Simpson indices measured species diversity. Coverage indicated the sequencing results’ comprehensiveness in representing the sample’s actual microbial composition, and the PD_whole_tree index assessed the overall diversity of the community. Alpha diversity analysis indicated increases in the Chao1, Ace, Shannon, Simpson, and PD_whole_tree indices across three dose groups, suggesting that HQGCD enhances mice’s diversity, community richness, and species count of the cecum microbiota. Further analysis of Beta diversity showed that the composition and structure of intestinal microbiota changed among the groups, with a trend of separation that was not obvious.

Among the top ten microbial groups with the highest abundance, *Firmicutes* and *Bacteroidetes* dominate the intestinal microbiota at the phylum level. The toxins produced by *Bacteroidetes* can induce proliferation of colonic epithelial cells and mucosal inflammation, in contrast, *Firmicutes* comprise many beneficial bacteria capable of reducing intestinal inflammation and regulating immune balance (Grice et al., 2009; Guo et al., 2022). The *F*/*B* value (*F* is the abundance of *Firmicutes*; *B* is the abundance of *Bacteroidetes*) in the intestinal microbiota of immunosuppressed mice decreases (Stojanov et al., 2020). An increase in the *F/B* ratio enhances the proportion of beneficial bacteria, increasing beneficial bacterial metabolites. This reduces the inflammatory response in the intestine and promotes positive regulation of immune function (So et al., 2021). This study found that HQGCD increased the *F/B* ratio in the cecum of mice. *Proteobacteria* and *Campylobacterota* are the dominant bacteria in the intestine. *Proteobacteria* and *Campylobacterota* are the main pathogens that cause diarrhea (Zhang et al., 2019). HQGCD could reduce the abundance of *Proteobacteria* and *Campylobacterota* in the cecum of mice. At the genus level, *unclassified_Muribaculaceae*shows shows a negative correlation with inflammatory factors TNF-α and IL-1β (Hu, 2022). *Unclassified_Lachnospiraceae* is enriched in the intestine and positively impacts host health. *Lachnospiraceae_NK4A136_group* is significantly upregulated and related to hematopoietic indicators (Wang et al., 2023). *Bacteroides* compete for available nutrients in their environment and degrade polysaccharides for their use, preventing other microorganisms from utilizing them (Zafar and Saier, 2021). *Alistipes* are believed to have a protective effect against several diseases, such as liver fibrosis, colitis, cancer during immunotherapy, and cardiovascular disease (Parker et al., 2020). *Lactobacillus* can stimulate the secretion of IgA and IgG by activating TLR2 expression in dendritic cells, B cells, and macrophages (Sakai et al., 2014). In this experiment, Cy administration led to an increase in the abundance of *unclassified_Muribaculaceae* and *Bacteroides*. Conversely, HQGCD treatment elevated the abundance of beneficial bacteria, including *unclassified Lachnospiraceae*, *Lachnospiraceae_NK4A136_group*, and *Lactobacillus*.

LEfSe analysis was conducted at each taxonomic level to identify species with a LDA score greater than 4, selecting those species for further investigation. LEfSe analysis revealed that Cy significantly altered the abundance of *Bacteroidota* and its subclass *Bacteroidia*, as well as *Erysipelatoclostridium* and *unclassified_Erysipelatoclostridium* within the *Erysipelatoclostridium* genus in the intestinal microbiota of mice, potentially leading to adverse health effects. Conversely, under HQGCD intervention, significant positive changes were observed in *Negativicutes*, including *Acidaminococcales*, *Acidaminococcaceae*, *Phascolarctobacterium*, *Ligilactobacillus*, *Oscillospiraceae*, and Lachnospiraceae. These taxa are known to be beneficial to animal health. These findings suggest that HQGCD can mitigate the harmful effects of Cy on the intestinal microbiota, promoting a shift towards a more beneficial microbial composition.

This experiment examined multiple indicators, demonstrating that HQGCD has a therapeutic effect on the immunosuppressed mice, with the HQGCD-H showing the most significant impact on the majority of the indicators.

## 5 Conclusion

In conclusion, HQGCD has obvious therapeutic effect on cyclophosphamide-induced immunosuppression mice, and increase the intestinal immunity ability of mice, which provides a basis for the further research and application of HQGCD.

## Data Availability

The data presented in the study are deposited in the https://www.ncbi.nlm.nih.gov/ repository, accession number PRJNA1089780.

## Supplementary material

SUPPLEMENTARY FIGURE S1 The standard curves of calycosin and glycyrrhizic acid.

## Abbreviations

Cy, cyclophosphamide; DP, CD4^+^CD8^+^ double-positive; DP IELs, DP T lymphocytes in IELs; HQGCD, Huangqi Gancao decoction; IEL, intestinal intraepithelial lymphocytes; MDA, malondialdehyde; OD, optical density; PCA, principal component analysis; sIgA, secretory immunoglobulin A; SOD, superoxide dismutase.
