# Supplementary figures and images for: Effects of Huangqi Gancao Decoction on intestinal immunity and microbiota in immunocompromised mice models
Source: Front Pharmacol. 2024 May 1;15:1390170. doi: 10.3389/fphar.2024.1390170 (PMC11097664; doi:10.3389/fphar.2024.1390170)

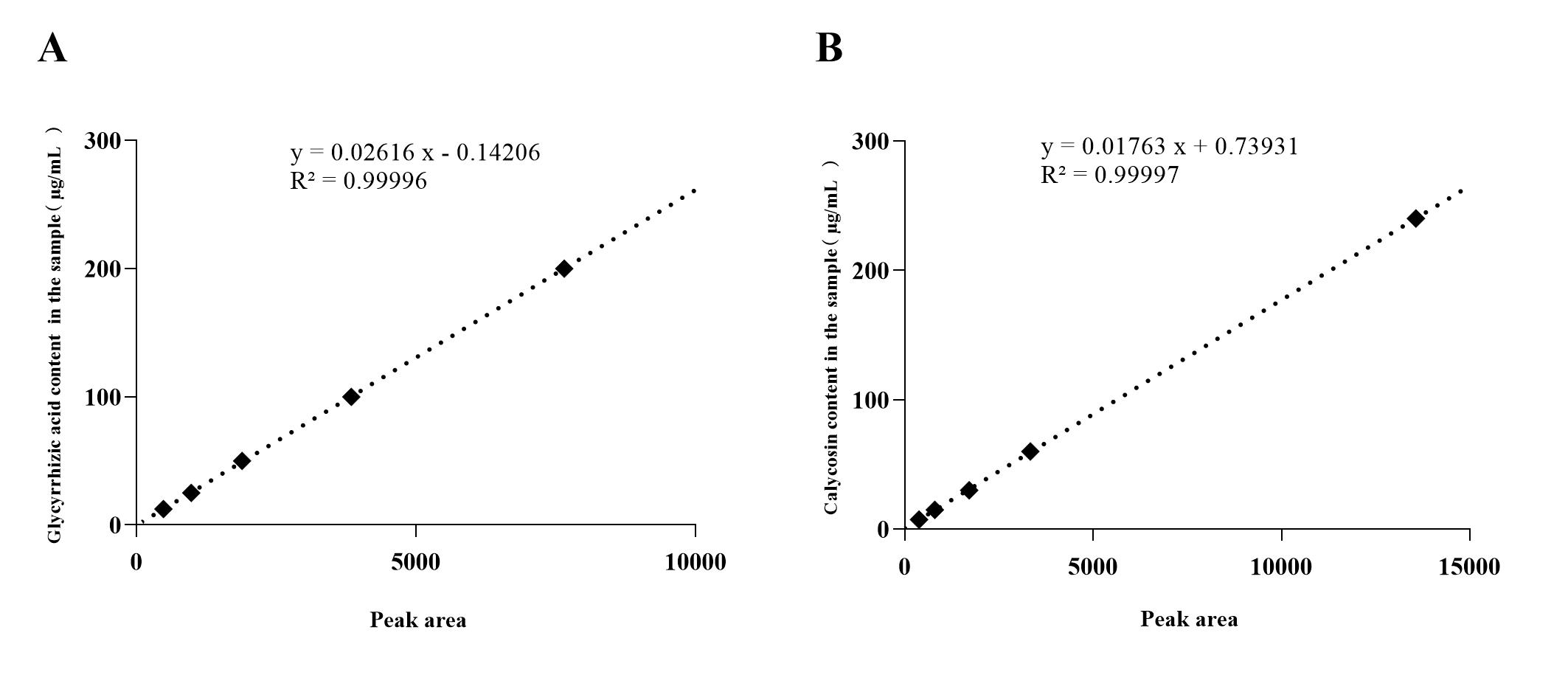

Supplement: Supplementary file 1 [file Image1.jpg]
